# Correlation between Liver Stiffness by Two-Dimensional Shear Wave Elastography and Waist Circumference in Japanese Local Citizens with Abdominal Obesity

**DOI:** 10.3390/jcm10091971

**Published:** 2021-05-04

**Authors:** Tomoki Miyoshi, Masahide Hamaguchi, Noriyuki Kitagawa, Yoshitaka Hashimoto, Michiaki Fukui

**Affiliations:** 1 Department of Endocrinology and Metabolism, Graduate School of Medical Science, Kyoto Prefectural University of Medicine, Kyoto 602-8566, Japan; tomokimi@koto.kpu-m.ac.jp (T.M.); y-hashi@koto.kpu-m.ac.jp (Y.H.); michiaki@koto.kpu-m.ac.jp (M.F.); 2 Department of Diabetology, Kameoka Municipal Hospital, Kameoka 621-8585, Japan; nori-kgw@koto.kpu-m.ac.jp

**Keywords:** liver stiffness, shear wave elastography, local citizens, abdominal obesity, diagnosis

## Abstract

Background: Various factors other than fibrosis could affect liver stiffness (LS), measured by two-dimensional shear wave elastography (2D-SWE). We aimed to clarify the factors affecting LS in local citizens. Methods: We performed a cross-sectional study among local citizens of a health checkup program. Abdominal obesity was defined as waist circumference ≥85 cm for men and ≥90 cm for women. We evaluated the correlation between LS by 2D-SWE (Aplio 500) and waist circumference with linear regression analyses. We selected the following items as variables in the multivariate analysis: waist circumference, sex, hypertension, diabetes, diagnostic components of metabolic syndrome, γ−glutamyl transpeptidase, total bilirubin, NAFLD fibrosis score, and an indicator of a fatty liver, evaluated ultrasonographically. Results: Overall, 345 individuals were included; 318 (181 men and 137 women; age, 63.4 years; waist circumference, 84.0 cm; LS, 5.79 kPa) were analyzed, 128 of whom had abdominal obesity and significantly higher LS than non-abdominally obese individuals. In the multivariate analysis, waist circumference was positively, independently, and significantly correlated with LS only in abdominally obese individuals. Conclusions: Liver stiffness by 2D-SWE could increase with increases in waist circumference in local citizens with abdominal obesity. Physicians should pay attention when assessing the LS of abdominally obese individuals.

## 1. Introduction

Liver fibrosis is an essential condition and predicts prognosis [1]. Although a liver biopsy is the gold standard for diagnosing liver fibrosis, it has several limitations, such as sampling error, interobserver discrepancy, and invasiveness [2,3]. Thus, alternative methods for diagnosing liver fibrosis have been investigated. Elastography can quantitatively measure tissue stiffness, an indicator of fibrosis, and elastography is applied to various diseases, including liver disease. Ultrasound elastography is now widely used to assess liver stiffness because of various advantages: cost-effectiveness, non-invasiveness, user-friendliness, and applicability at the bedside [4].

Transient elastography is a kind of ultrasound elastography that became clinically available the earliest and most widely used than other ultrasound elastography [5]. Liver stiffness by transient elastography is well correlated with histologically diagnosed liver fibrosis [6,7]. However, transient elastography tends to fail to detect liver stiffness in the presence of ascites or thick-subcutaneous fat. [8] Recently developed, two-dimensional shear wave elastography (2D-SWE) can measure liver stiffness in real-time by overlaying a region of interest on conventional B-mode imaging, improving the ability to detect liver stiffness [9]. Two-dimensional shear wave elastography is becoming widely used and is recommended for use in the evaluation of liver fibrosis by some guidelines [8,10]. Two-dimensional shear wave elastography can be used to rule out liver fibrosis in local citizens, especially in those with metabolic syndrome, since metabolic syndrome is associated with higher liver stiffness [11,12].

However, liver stiffness may differ from actual liver fibrosis in some conditions. Breathing, cardiac beats, liver congestion, liver inflammation, cholestasis, and a fatty liver have all been reported to affect liver stiffness [4]. Previous 2D-SWE studies have been mainly performed in patients with chronic liver diseases, such as viral hepatitis and non-alcoholic fatty liver disease (NAFLD). Hence, the factors influencing liver stiffness by 2D-SWE in local citizens, a population without overt liver fibrosis, remain unclear. If liver stiffness is high in local citizens, physicians need to decide whether more invasive examinations, including a liver biopsy, should be performed or not. Nonetheless, the evidence supporting this judgment is lacking. Therefore, we examined the factors affecting liver stiffness by 2D-SWE in local citizens.

## 2. Materials and Methods

### 2.1. Study Design, Participants, and Ethics

We held a cross-sectional study of individuals who participated in a health checkup program held at Kameoka Municipal Hospital in Japan between May 2017 and March 2019. Participants were asymptomatic individuals who voluntarily participated in the program to check their health. Those who had ascites found by abdominal ultrasonography, heart failure, the positive result of a hepatitis B surface antigen test or a hepatitis C virus antibody test, the medical history of alcoholic liver disease, or unreliable liver stiffness were excluded. Those who did not undergo liver stiffness measurement were also excluded. This study was held under the Declaration of Helsinki in 1975 and approved by the Ethics Committee of Kameoka Municipal Hospital (ID 30-3). This study’s consent was obtained by the opt-out approach based on the Ethical Guidelines for Medical and Health Research Involving Human Subjects in Japan.

### 2.2. Data Collection and Measurement of Liver Stiffness

Standardized self-administered questionnaires and medical interviews were used to acquire participants’ age, sex, and comorbidities. The following data were obtained from the results of the health checkup program: body mass index (BMI); waist circumference; systolic blood pressure; diastolic blood pressure; white blood cell and platelet counts; fasting blood glucose, hemoglobin A1c, total bilirubin, aspartate aminotransferase (AST), alanine aminotransferase (ALT), lactate dehydrogenase, alkaline phosphatase, γ-glutamyl transpeptidase, albumin, blood urea nitrogen, C-reactive protein, total cholesterol, triglycerides, high-density lipoprotein (HDL) cholesterol, and low-density lipoprotein cholesterol; estimated glomerular filtration rate; liver stiffness; the ultrasonographic findings of a fatty liver (UFL) score, indicating a fatty liver’s degree; the NAFLD fibrosis score and the Fibrosis-4 (FIB-4) index, indicating a degree of liver fibrosis. The participants were instructed to fast overnight before undergoing blood tests and abdominal ultrasonography.

**Figure 1:**
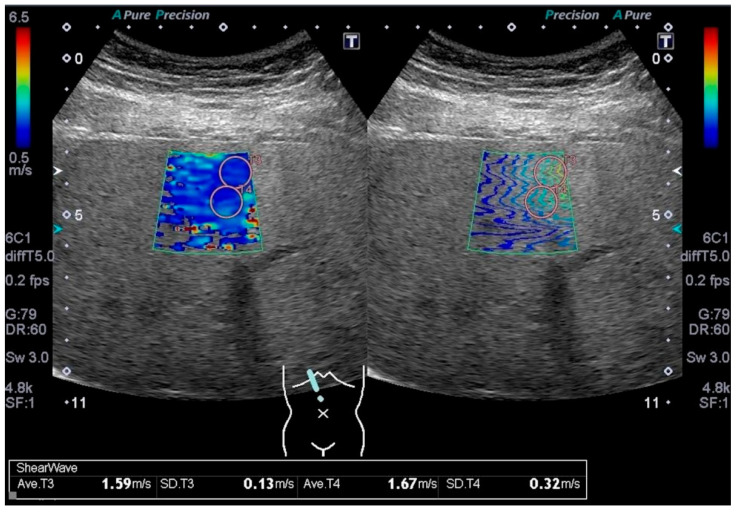
An example image of 2D-SWE in a participant with increased waist circumference (111 cm).

Trained ultrasonographers performed abdominal ultrasonography. Liver stiffness was evaluated using 2D-SWE (Aplio 500, Canon Medical Systems Corporation, Otawara, Japan) (Figure 1) according to the guideline of the World Federation of Ultrasound in Medicine and Biology for liver elastography [10]. Liver stiffness was evaluated by 2D-SWE five to seven times, and we regarded liver stiffness with an interquartile range/median value > 30% as unreliable.

### 2.3. Degree of Fatty Liver, UFL Score

A fatty liver’s degree was semi-quantitatively evaluated using a scoring system with ultrasonographic findings of a fatty liver, the reliability of which has been validated previously [13]. Briefly, a fatty liver’s degree was scored between 0 and 6 points depending on the following ultrasonographic findings: bright liver, hepatorenal echo contrast, deep attenuation, and vessel blurring. The score was called the UFL score in this study. The higher the score, it indicated the more severe the fatty liver was. We used a UFL score of 2 or higher as the cutoff level for fatty liver disease.

### 2.4. Definition of Hypertension, Diabetes, Dyslipidemia, Fatty Liver Disease, BMI, NAFLD Fibrosis Score, and FIB-4 Index

Hypertension was defined as when a patient was either diagnosed with or under medical treatment for hypertension. Diabetes was defined as when the patient had both fasting blood glucose ≥ 126 mg/dL and hemoglobin A1c ≥ 6.5%, or under medical treatment for diabetes. Dyslipidemia was defined as when the patients were under medical treatment for dyslipidemia or having at least one of the following: total cholesterol ≥ 220 mg/dL, triglycerides ≥ 150 mg/dL, HDL cholesterol ≤ 39 mg/dL, or low-density lipoprotein cholesterol ≥ 140 mg/dL. Fatty liver disease was defined as the patient having a UFL score of 2 or higher. BMI = body weight (kg)/height (m^2^). NAFLD fibrosis score = −1.675 + 0.037 × age (years) + 0.094 × BMI (kg/m^2^) + 1.13 × IFG/diabetes (yes = 1, no = 0) + 0.99 × AST/ALT ratio − 0.013 × platelet (×10^9^/L) − 0.66 × albumin (g/dL) [14]. FIB-4 index = (age (years) × AST (IU/L))/(platelet count (10^9^/L) × (ALT (IU/L))^1/2^) [15].

### 2.5. Participant Subgroups

The participants were divided into two subgroups for the analysis: those with abdominal obesity (abdominally obese group) and those without abdominal obesity (non-abdominally obese group). Abdominal obesity was defined as waist circumference ≥85 cm for men and ≥90 cm for women based on the Japanese diagnostic criteria of metabolic syndrome [16].

### 2.6. Statistical Analysis

Categorical variables were presented as percentages (absolute numbers), and continuous variables were presented as means (standard deviations). We performed Pearson’s chi-square tests for comparing categorical variables and Student’s t-tests for comparing continuous variables between the subgroups. Two-sided tests were performed to calculate the *p*-values; *p*-values < 0.05 were considered to indicate statistical significance. The Windows version of JMP 14 (SAS Institute Inc., Cary, NC, USA, version 14.3.0) was used as the analysis software. Univariate and multivariate linear regression analyses were performed to see the correlations between liver stiffness and variables.

## 3. Results

**Figure 2:**
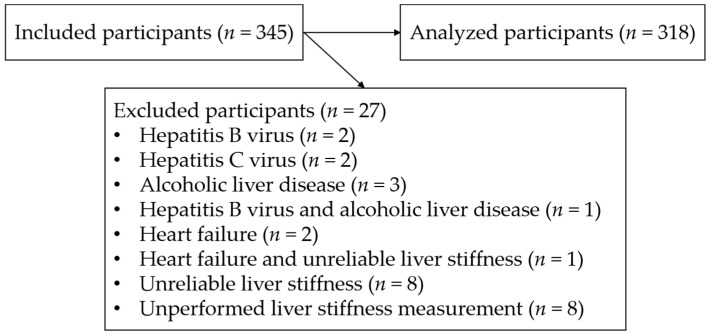
Study flowchart.

A total of 346 individuals participated in the health checkup program, and one man requested to resign from this study. The remaining 345 people were included, of whom, 27 were excluded for the following reasons: hepatitis B virus (*n* = 2), hepatitis C virus (*n* = 2), alcoholic liver disease (*n* = 3), both hepatitis B virus and alcoholic liver disease (*n* = 1), heart failure (*n* = 2), both heart failure and unreliable liver stiffness (*n* = 1), unreliable liver stiffness (*n* = 8), and unperformed liver stiffness measurement (*n* = 8). Finally, 318 participants were analyzed (Figure 2).

Overall, 57% of the participants were male, with a mean age of 63.4 ± 12.2 years. Comorbidities included hypertension (41%), diabetes (13%), dyslipidemia (49%), and fatty liver disease (41%). Liver stiffness was 5.79 ± 1.11 kPa, the NAFLD fibrosis score was −1.333 ± 1.217, and the FIB-4 index was 1.60 ± 0.67, respectively. No one had 15 kPa or more of liver stiffness, the cutoff value of 2D-SWE used in this study for diagnosing F4 of the METAVIR score [4]. A total of 46% of the participants had an NAFLD fibrosis score < −1.455; 6% had an NAFLD fibrosis score > 0.676; 36% had an FIB-4 index < 1.3; and 7% an FIB-4 index ≥ 2.67, respectively. Approximately 40% of the participants had abdominal obesity. The abdominally obese group had a higher proportion of males (*p* < 0.001) and a higher prevalence of hypertension (*p* < 0.001), diabetes (*p* = 0.040), and fatty liver disease (*p* < 0.001) than the non-abdominally obese group.

The following continuous variables were significantly higher in the abdominally obese group than in the non-abdominally obese group: liver stiffness, BMI, waist circumference, systolic blood pressure, fasting blood glucose, ALT, γ-glutamyl transpeptidase, triglycerides, the UFL score, and the NAFLD fibrosis score. The HDL cholesterol levels and the proportion of participants with an NAFLD fibrosis score < −1.455 were significantly lower in the abdominally obese group than in the non-abdominally obese group. There were no significant differences in age, platelet count, and the FIB-4 index between the subgroups (Table 1).

### Correlation of Liver Stiffness with Waist Circumference

In the univariate analysis, overall, liver stiffness was significantly correlated with waist circumference (*β* = 0.28, *p* < 0.001); BMI (*β* = 0.26, *p* < 0.001); systolic blood pressure (*β* = 0.16, *p* = 0.004); diastolic blood pressure (*β* = 0.16, *p* = 0.004); white blood cell count (*β* = 0.11, *p* = 0.049); fasting blood glucose (*β* = 0.11, *p* = 0.046), total bilirubin (*β* = −0.15, *p* = 0.006), AST (*β* = 0.12, *p* = 0.04), ALT (*β* = 0.13, *p* = 0.018), total cholesterol (*β* = −0.11, *p* = 0.041), triglycerides (*β* = 0.16, *p* = 0.005), HDL cholesterol (*β* = −0.18, *p* < 0.001); and the UFL score (*β* = 0.22, *p* < 0.001). The NAFLD fibrosis score and the FIB-4 index were not correlated with liver stiffness.

In the abdominally obese group, liver stiffness was significantly correlated with waist circumference (*β* = 0.26, *p* = 0.003), BMI (*β* = 0.28, *p* = 0.001), total bilirubin (*β* = −0.22, *p* = 0.012), and the UFL score (*β* = 0.21, *p* = 0.019), but the NAFLD fibrosis score and the FIB-4 index were not positively correlated with liver stiffness. In the non-abdominally obese group, liver stiffness was significantly correlated with age (*β* = 0.18, *p* = 0.012), the NAFLD fibrosis score (*β* = 0.18, *p* = 0.013), and the FIB-4 index (*β* = 0.18, *p* = 0.014), but waist circumference, BMI, and the UFL score were not correlated with liver stiffness (Table 2).

In the multivariate analysis with the NAFLD fibrosis score, liver stiffness was significantly correlated with waist circumference; *β* was 0.16 (*p* = 0.036) in all participants, whereas *β* was 0.22 (*p* = 0.036) in the abdominally obese group. The following items were not significantly correlated with liver stiffness both in all participants and in the abdominally obese group: sex, presence of hypertension or diabetes, systolic blood pressure, fasting blood glucose, triglycerides, HDL cholesterol, the FIB-4 index, and the UFL score. No variables were associated with liver stiffness in the non-abdominally obese group (Table 3). A similar result was observed in the multivariate analysis with the FIB-4 index, though the *p*-value of waist circumference in the abdominally obese group was 0.056 (Table 4). The variance inflation factors of all variables in both multivariate analyses were less than 2.4.

## 4. Discussion

Our study showed that liver stiffness by 2D-SWE increased with increases in waist circumference in abdominally obese individuals after adjustment for the confounding of liver fibrosis and steatosis. Bazerbachi et al. performed a meta-analysis with multivariate linear regression analysis in apparently healthy individuals. They found that liver stiffness by transient elastography was positively and independently correlated with waist circumference [17]. Our study provides a novel finding that a positive correlation of liver stiffness by 2D-SWE with waist circumference exists in abdominally obese individuals but not in non-abdominally obese individuals. Moreover, we found a possibility that such a correlation is beyond liver fibrosis and steatosis.

We think that physical compression of the liver by visceral fat can increase liver stiffness in the absence of liver fibrosis in abdominally obese individuals. Since excessive external compression of the liver by an ultrasound probe has been reported to increase liver stiffness by 2D-SWE [18], it is reasonable to think that excessive internal compression of the liver also increases liver stiffness. It is well known that waist circumference is correlated with the visceral fat area [19]. In the presence of abdominal obesity, the abdominal cavity is filled with visceral fat, and a further increase in visceral fat may compress the liver, leading to increased liver stiffness. Contrastingly, in the absence of abdominal obesity, visceral fat growth does not necessarily increase liver stiffness because there is room for visceral fat to grow without forcefully compressing the liver. We believe that this explains why waist circumference did not correlate with liver stiffness in the non-abdominally obese group.

However, the correlation between liver stiffness by 2D-SWE and waist circumference in abdominally obese individuals may simply derive from liver fibrosis, not from liver compression. We did not histologically examine liver fibrosis because performing liver biopsies on asymptomatic-local citizens was ethically challenging. We instead used the NAFLD fibrosis score and the FIB-4 index for evaluating liver fibrosis. As for the FIB-4 index, in Japanese NAFLD patients diagnosed with liver biopsies, Sumida et al. reported that a negative predictive value for liver-fibrosis stage ≥ F3 of the METAVIR score was 94% when the FIB-4 index cutoff value was set at 3.25 [20]. A total of 98% of the participants in this study had an FIB-4 index < 3.25, suggesting that we had included few participants with advanced liver fibrosis in this study.

Nonetheless, non-invasive scoring systems for evaluating liver fibrosis, such as the NAFLD fibrosis score and the FIB-4 index, perform much better in excluding advanced liver fibrosis rather than diagnosing it [21]. Additionally, it has not been proved that non-invasive scoring systems can exclude mild to moderate liver fibrosis, meaning that we may have included those with mild to moderate liver fibrosis in this study. Moreover, in the multivariate analysis—not with the NAFLD fibrosis score but with the FIB-4 index—the p-value of waist circumference in the abdominally obese group was slightly above 0.05. This result means that our study’s sample size might not have been enough to detect the correlation between liver stiffness by 2D-SWE and waist circumferences after correction by several covariates. Hence, the correlation between liver stiffness by 2D-SWE and waist circumference may come from liver fibrosis. Further studies are needed to confirm whether it is genuine that liver stiffness by 2D-SWE can increase beyond liver fibrosis.

The policy for assessing liver fibrosis in local citizens with high liver stiffness by 2D-SWE has not been established. Physicians should consider performing a liver biopsy when liver stiffness is increased. Still, it is challenging to perform liver biopsies on all those with high liver stiffness, considering liver biopsy disadvantages, such as invasiveness [2]. We think that serum biomarkers might be more suitable for further screening. Though no serum biomarkers have been validated in local citizens, the findings in those with NAFLD are thought to be useful since local citizens partially include those with NAFLD. The areas under the receiver operating characteristic curve (AUROC) of Wisteria floribunda agglutinin-positive Mac-2-binding protein [22,23] and type IV collagen 7s [23,24] for detecting liver fibrosis have been reported to be high in those with NAFLD. We think that assessing these serum biomarkers before performing a liver biopsy is necessary.

This study has some limitations. As for a non-invasive scoring system, Ampuero et al. recently developed the Hepamet fibrosis score (HFS). They reported that HFS had a greater AUROC than the NAFLD fibrosis score and the FIB-4 index in NAFLD patients [25]. Ballestri et al. also reported that the Hepamet fibrosis score had the greatest AUROC in NAFLD patients among other non-invasive scoring systems [21]. Though we cannot calculate HFS since we had not tested participants’ serum insulin levels, it would have been better to add HFS to our analysis to more firmly exclude those with liver fibrosis.

We examined a degree of a fatty liver not with histology but with the UFL score. Though the UFL score’s reliability as an indicator for a fatty liver has been proved in one report [13], other reports have not validated the UFL score. Thus, the assessment of a fatty liver might not be enough, which might affect our results. As a non-invasive assessment method for a fatty liver, the Controlled Attenuation Parameter (CAP) might have been more suitable than the UFL score because the correlation between the CAP and a fatty liver has been well proved. However, the CAP is acquired with transient elastography, not with 2D-SWE. It was difficult to perform both 2D-SWE and transient elastography on one participant. Moreover, the Ultrasonographic Fatty Liver Indicator (US-FLI) has been reported to be correlated with the severity of liver steatosis assessed with histology or the CAP as a non-invasive and semi-quantitative assessment for a fatty liver with ultrasonography [26,27]. It may have been more appropriate to use US-FLI than the UFL score from the perspective of validation.

Trained ultrasonographers evaluated LS and the UFL score in this study. However, we had not made interobserver agreements of these parameters. The lack of interobserver agreements might have reduced the reproducibility of our study.

Since the present study’s participants consisted of individuals who voluntarily participated in a health checkup program, this study may have had a selection bias, unlike a population-based study. Actually, the participant’s average age was higher than the average in Japan. However, the prevalence of hypertension and diabetes in the present study was almost the same as the National Health and Nutrition Survey of Japan (2018) [28]. Moreover, we excluded three participants with hepatitis B (0.9%) and two participants with hepatitis C (0.6%) from this study, which also showed no discrepancy with the prevalence of these liver diseases in Japan [29]. Consequently, it was reasonable to think that this study’s participants were representatives of local citizens.

Colli et al. reported that liver congestion due to right heart failure was a confounding factor affecting liver stiffness by ultrasound elastography [30]. In this study, echocardiography was not performed in all participants. However, those with a past medical history of heart failure or those suspected to have heart failure based on chest X-ray were excluded. Hence, we believe that relatively few participants had liver congestion.

## 5. Conclusions

Liver stiffness by 2D-SWE could increase with an increase in waist circumference in local citizens with abdominal obesity. We think that this correlation might cause the overestimation of liver fibrosis. In such individuals, physicians should not diagnose liver fibrosis only based on high liver stiffness. Physicians should additionally perform low-invasive examinations, including Wisteria floribunda agglutinin-positive Mac-2 binding protein or type IV collagen, before a liver biopsy.

## Figures and Tables

**Table 1:** Participant characteristics.

Characteristic	All Participants	Abdominally Obese Group	Non-Abdominally Obese Group	*p*-Value
	*n* = 318	*n* = 128	*n* = 190	
Male sex, % (*n*)	57 (181)	86 (110)	37 (71)	<0.001
Hypertension, % (*n*)	41 (130)	63 (81)	26 (49)	<0.001
Diabetes, % (*n*)	13 (41)	18 (23)	9 (18)	0.040
Dyslipidemia, % (*n*)	49 (157)	47 (60)	51 (97)	0.49
Fatty liver disease, % (*n*)	41 (129)	63 (81)	25 (48)	<0.001
Age (years)	63.4 (12.2)	64.8 (11.4)	62.6 (12.7)	0.11
Body mass index (kg/m^2^)	22.7 (3.5)	25.5 (3.0)	20.9 (2.4)	<0.001
Waist circumference (cm)	84.0 (10.0)	93.4 (6.4)	77.7 (6.4)	<0.001
Systolic blood pressure (mmHg)	131 (18)	137 (18)	127 (17)	<0.001
Diastolic blood pressure (mmHg)	81 (12)	85 (12)	77 (11)	<0.001
White blood cell (×10^3^/μL)	5.2 (1.6)	5.7 (1.8)	4.9 (1.4)	<0.001
Platelet (×10^4^/μL)	21.3 (4.8)	21.1 (5.0)	21.4 (4.7)	0.52
Fasting blood glucose (mg/dL)	105 (17)	109 (17)	103 (16)	<0.001
Hemoglobin A1c (%)	5.81 (0.57)	5.92 (0.65)	5.74 (0.49)	0.004
Total bilirubin (mg/dL)	0.98 (0.38)	0.94 (0.37)	1.01 (0.38)	0.12
Aspartate aminotransferase (IU/L)	22 (7)	23 (8.2)	22 (6)	0.097
Alanine aminotransferase (IU/L)	21 (13)	26 (17)	18 (8)	<0.001
Lactate dehydrogenase (IU/L)	203 (60)	206 (59)	202 (60)	0.55
Alkaline phosphatase (IU/L)	177 (30)	179 (32)	175 (28)	0.27
γ-glutamyl transpeptidase (IU/L)	35 (47)	46 (64)	28 (29)	<0.001
Albumin (g/dL)	4.30 (0.25)	4.27 (0.25)	4.32 (0.25)	0.11
Blood urea nitrogen (mg/dL)	15.1 (3.8)	15.8 (4.0)	14.6 (3.7)	0.004
Estimated glomerular filtration rate (ml/min/1.73m^2^)	67.4 (14.3)	64.7 (14.0)	69.2 (14.2)	0.006
C-reactive protein (mg/dL)	0.13 (0.42)	0.17 (0.30)	0.10 (0.49)	0.15
Total cholesterol (mg/dL)	204 (32)	197 (32)	209 (30)	<0.001
Triglycerides (mg/dL)	106 (68)	136 (87)	86 (40)	<0.001
High-density lipoprotein cholesterol (mg/dL)	67 (18)	56 (13)	75 (17)	<0.001
Low-density lipoprotein cholesterol (mg/dL)	123 (28)	123 (28)	123 (27)	0.89
UFL score	1.2 (1.5)	2.0 (1.7)	0.7 (1.0)	<0.001
NAFLD fibrosis score	−1.333 (1.217)	−1.013 (1.217)	−1.549 (1.172)	<0.001
NAFLD fibrosis score < −1.455, % (*n*)	46 (147)	33 (42)	55 (105)	<0.001
NAFLD fibrosis score > 0.676, % (*n*)	6 (18)	7 (9)	5 (9)	0.39
FIB-4 index	1.60 (0.67)	1.56 (0.63)	1.62 (0.70)	0.37
FIB-4 index < 1.3, % (*n*)	36 (114)	38 (49)	34 (65)	0.46
FIB-4 index ≥ 2.67, % (*n*)	7 (21)	5 (6)	8 (15)	0.26
Liver stiffness (kPa)	5.79 (1.11)	6.13 (1.38)	5.56 (0.80)	<0.001

Categorical variables were presented as percentages (absolute numbers). Continuous variables were presented as mean (standard deviations). Between the subgroups, Pearson’s chi-square test was performed for comparing categorical variables, and Student’s t-test was performed to compare continuous variables. A two-sided test was performed to calculate the *p*-value. The UFL score was a scoring system for semi-quantitatively evaluating a fatty liver. A fatty liver’s degree was scored between 0 and 6 points depending on the following ultrasonographic findings of a fatty liver: bright liver, hepatorenal echo contrast, deep attenuation, and vessel blurring. The higher the score, it indicated the more severe the fatty liver was. Fatty liver disease was defined as a UFL score of 2 or higher. UFL, ultrasonographic findings of a fatty liver; NAFLD, non-alcoholic fatty liver disease; FIB-4, fibrosis-4.

**Table 2:** The correlations between liver stiffness and each variable, univariate linear regression analysis.

Variable	All Participants		Abdominally Obese Group		Non-Abdominally Obese Group	
	*β*	*p*-value	*β*	*p*-value	*β*	*p*-value
Age	0.04	0.45	−0.13	0.14	0.18	0.012
Body mass index	0.26	<0.001	0.28	0.001	−0.08	0.25
Waist circumference	0.28	<0.001	0.26	0.003	0.00	1.0
Systolic blood pressure	0.16	0.004	0.09	0.32	0.12	0.10
Diastolic blood pressure	0.16	0.004	0.15	0.091	0.02	0.80
White blood cell	0.11	0.049	0.13	0.13	−0.06	0.40
Platelet	0.06	0.28	0.16	0.072	−0.03	0.68
Fasting blood glucose	0.11	0.046	0.04	0.65	0.11	0.15
Hemoglobin A1c	0.09	0.12	0.05	0.57	0.05	0.50
Total bilirubin	−0.15	0.006	−0.22	0.012	−0.05	0.50
Aspartate aminotransferase	0.12	0.038	0.13	0.13	0.04	0.63
Alanine aminotransferase	0.13	0.018	0.10	0.24	−0.04	0.61
Lactate dehydrogenase	0.06	0.33	0.08	0.37	0.02	0.83
Alkaline phosphatase	0.03	0.55	−0.02	0.84	0.07	0.35
γ-glutamyl transpeptidase	0.10	0.062	0.08	0.36	0.01	0.94
Albumin	−0.06	0.27	−0.03	0.71	−0.05	0.46
Blood urea nitrogen	0.02	0.68	−0.11	0.22	0.10	0.19
Estimated glomerular filtration rate	0.00	0.97	0.11	0.22	−0.04	0.59
C-reactive protein	0.03	0.60	0.07	0.45	−0.03	0.67
Total cholesterol	−0.11	0.041	−0.07	0.46	−0.08	0.30
Triglycerides	0.16	0.005	0.12	0.18	−0.04	0.57
High-density lipoprotein cholesterol	−0.18	0.002	−0.12	0.19	−0.01	0.94
Low-density lipoprotein cholesterol	−0.06	0.26	−0.06	0.53	−0.08	0.29
UFL score	0.22	<0.001	0.21	0.019	−0.04	0.61
NAFLD fibrosis score	0.07	0.21	−0.12	0.19	0.18	0.013
FIB-4 index	−0.02	0.75	−0.18	0.042	0.18	0.014

The least-squares method was used to calculate standardized regression coefficients, *β*, and *p*-values. The UFL score was a grading system for semi-quantitatively evaluating a fatty liver. A fatty liver’s degree was scored between 0 and 6 points depending on the following ultrasonographic findings of a fatty liver: bright liver, hepatorenal echo contrast, deep attenuation, and vessel blurring. The higher the score, it indicated the more severe the fatty liver was. UFL, ultrasonographic findings of a fatty liver; NAFLD, non-alcoholic fatty liver disease; FIB-4, fibrosis-4.

**Table 3:** The correlation of liver stiffness with waist circumference after adjusting potential covariates, multivariate linear regression analysis with the NAFLD fibrosis score.

Variable	All Participants		Abdominally Obese Group		Non-Abdominally Obese Group	
	*β*	*p*-Value	*β*	*p*-Value	*β*	*p*-Value
Waist circumference	0.16	0.036	0.22	0.036	−0.03	0.68
Male sex	0.09	0.15	0.13	0.16	0.07	0.41
Hypertension	0.08	0.28	0.05	0.61	0.13	0.18
Diabetes	0.02	0.75	−0.03	0.79	0.08	0.46
Systolic blood pressure	0.02	0.74	0.00	0.99	0.04	0.65
Fasting blood glucose	−0.01	0.95	0.00	0.97	−0.04	0.69
Triglycerides	−0.01	0.93	−0.06	0.61	−0.08	0.38
High-density lipoprotein cholesterol	0.04	0.57	−0.01	0.91	0.00	0.99
γ-glutamyl transpeptidase	0.03	0.57	0.02	0.86	0.01	0.88
Total bilirubin	−0.14	0.015	−0.20	0.025	−0.06	0.44
UFL score	0.09	0.21	0.12	0.27	−0.02	0.82
NAFLD fibrosis score	0.00	0.99	−0.07	0.53	0.10	0.24

The least-squares method was used to calculate the standardized partial regression coefficients, *β*, and *p*-values. The UFL score was a grading system for semi-quantitatively evaluating a fatty liver. A fatty liver’s degree was scored between 0 and 6 points depending on the following ultrasonographic findings of a fatty liver: bright liver, hepatorenal echo contrast, deep attenuation, and vessel blurring. The higher the score, it indicated the more severe the fatty liver was. UFL, ultrasonographic findings of a fatty liver; NAFLD, non-alcoholic fatty liver disease.

**Table 4:** The correlation of liver stiffness with waist circumference after adjusting potential covariates, multivariate linear regression analysis with the FIB-4 index.

Variable	All participants		Abdominally Obese Group		Non-Abdominally Obese Group	
	*β*	*p*-Value	*β*	*p*-Value	*β*	*p*-Value
Waist circumference	0.16	0.039	0.20	0.056	−0.02	0.81
Male sex	0.09	0.14	0.13	0.18	0.06	0.51
Hypertension	0.08	0.27	0.07	0.51	0.13	0.16
Diabetes	0.03	0.73	−0.03	0.78	0.08	0.46
Systolic blood pressure	0.02	0.73	0.00	0.98	0.03	0.77
Fasting blood glucose	−0.01	0.93	−0.02	0.86	−0.01	0.90
Triglycerides	−0.01	0.92	−0.06	0.60	−0.07	0.44
High-density lipoprotein cholesterol	0.04	0.56	−0.01	0.90	0.00	0.97
γ-glutamyl transpeptidase	0.03	0.56	0.02	0.80	0.00	0.97
Total bilirubin	−0.14	0.015	−0.20	0.026	−0.06	0.44
UFL score	0.08	0.22	0.11	0.31	−0.03	0.74
FIB-4 index	−0.01	0.84	−0.10	0.29	0.11	0.18

The least-squares method was used to calculate the standardized partial regression coefficients, *β,* and *p*-values. The UFL score was a grading system for semi-quantitatively evaluating a fatty liver. A fatty liver’s degree was scored between 0 and 6 points depending on the following ultrasonographic findings of a fatty liver: bright liver, hepatorenal echo contrast, deep attenuation, and vessel blurring. The higher the score, it indicated the more severe the fatty liver was. UFL, ultrasonographic findings of a fatty liver; FIB-4, fibrosis-4.

## Data Availability

The data that support the findings of this study are available from the corresponding author upon reasonable request.

## References

[1] Angulo P., Kleiner D.E., Dam-Larsen S., Adams L.A., Bjornsson E.S., Charatcharoenwitthaya P., Mills P.R., Keach J.C., Lafferty H.D., Stahler A. (2015). Liver fibrosis, but no other histologic features, is associated with long-term outcomes of patients with nonalcoholic fatty liver disease. Gastroenterology.

[2] Bravo A.A., Sheth S.G., Chopra S. (2001). Liver Biopsy. N. Engl. J. Med..

[3] Regev A., Berho M., Jeffers L.J., Milikowski C., Molina E.G., Pyrsopoulos N.T., Feng Z.Z., Reddy K.R., Schiff E.R. (2002). Sampling error and intraobserver variation in liver biopsy in patients with chronic HCV infection. Am. J. Gastroenterol..

[4] Sigrist R.M.S., Liau J., El Kaffas A., Chammas M.C., Willmann J.K. (2017). Ultrasound elastography: Review of techniques and clinical applications. Theranostics.

[5] Garra B.S. (2015). Elastography: History, principles, and technique comparison. Abdom. Imaging.

[6] Castera L., Friedrich-Rust M., Loomba R. (2019). Noninvasive Assessment of Liver Disease in Patients With Nonalcoholic Fatty Liver Disease. Gastroenterology.

[7] Afdhal N.H., Bacon B.R., Patel K., Lawitz E.J., Gordon S.C., Nelson D.R., Challies T.L., Nasser I., Garg J., Wei L.J. (2015). Accuracy of fibroscan, compared with histology, in analysis of liver fibrosis in patients with hepatitis B or C: A united states multicenter study. Clin. Gastroenterol. Hepatol..

[8] Castera L., Yuen Chan H.L., Arrese M., Afdhal N., Bedossa P., Friedrich-Rust M., Han K.H., Pinzani M. (2015). EASL-ALEH Clinical Practice Guidelines: Non-invasive tests for evaluation of liver disease severity and prognosis. J. Hepatol..

[9] Ferraioli G., Tinelli C., Dal Bello B., Zicchetti M., Filice G., Filice C. (2012). Accuracy of real-time shear wave elastography for assessing liver fibrosis in chronic hepatitis C: A pilot study. Hepatology.

[10] Ferraioli G., Filice C., Castera L., Choi B.I., Sporea I., Wilson S.R., Cosgrove D., Dietrich C.F., Amy D., Bamber J.C. (2015). WFUMB guidelines and recommendations for clinical use of ultrasound elastography: Part 3: Liver. Ultrasound Med. Biol..

[11] Roulot D., Czernichow S., Le Clésiau H., Costes J.L., Vergnaud A.C., Beaugrand M. (2008). Liver stiffness values in apparently healthy subjects: Influence of gender and metabolic syndrome. J. Hepatol..

[12] Kumar M., Sharma P., Garg H., Kumar R., Bhatia V., Sarin S.K. (2011). Transient elastographic evaluation in adult subjects without overt liver disease: Influence of alanine aminotransferase levels. J. Gastroenterol. Hepatol..

[13] Hamaguchi M., Kojima T., Itoh Y., Harano Y., Fujii K., Nakajima T., Kato T., Takeda N., Okuda J., Ida K. (2007). The severity of ultrasonographic findings in nonalcoholic fatty liver disease reflects the metabolic syndrome and visceral fat accumulation. Am. J. Gastroenterol..

[14] Sterling R.K., Lissen E., Clumeck N., Sola R., Correa M.C., Montaner J., Sulkowski M.S., Torriani F.J., Dieterich D.T., Thomas D.L. (2006). Development of a simple noninvasive index to predict significant fibrosis in patients with HIV/HCV coinfection. Hepatology.

[15] Angulo P., Hui J.M., Marchesini G., Bugianesi E., George J., Farrell G.C., Enders F., Saksena S., Burt A.D., Bida J.P. (2007). The NAFLD fibrosis score: A noninvasive system that identifies liver fibrosis in patients with NAFLD. Hepatology.

[16] Definition and the diagnostic standard for metabolic syndrome (2005). Committee to Evaluate Diagnostic Standards for Metabolic Syndrome. Nihon Naika Gakkai Zasshi..

[17] Bazerbachi F., Haffar S., Wang Z., Cabezas J., Arias-Loste M.T., Crespo J., Darwish-Murad S., Ikram M.A., Olynyk J.K., Gan E. (2019). Range of Normal Liver Stiffness and Factors Associated With Increased Stiffness Measurements in Apparently Healthy Individuals. Clin. Gastroenterol. Hepatol..

[18] Naganuma H., Ishida H., Uno A., Nagai H., Kuroda H., Ogawa M. (2020). Diagnostic problems in two-dimensional shear wave elastography of the liver. World J. Radiol..

[19] Hiuge-Shimizu A., Kishida K., Funahashi T., Ishizaka Y., Oka R., Okada M., Suzuki S., Takaya N., Nakagawa T., Fukui T. (2012). Absolute value of visceral fat area measured on computed tomography scans and obesity-related cardiovascular risk factors in large-scale Japanese general population (the VACATION-J study). Ann. Med..

[20] Sumida Y., Yoneda M., Hyogo H., Itoh Y., Ono M., Fujii H., Eguchi Y., Suzuki Y., Aoki N., Kanemasa K. (2012). Validation of the FIB4 index in a Japanese nonalcoholic fatty liver disease population. BMC Gastroenterol..

[21] Ballestri S., Mantovani A., Baldelli E., Lugari S., Maurantonio M., Nascimbeni F., Marrazzo A., Romagnoli D., Targher G., Lonardo A. (2021). Liver Fibrosis Biomarkers Accurately Exclude Advanced Fibrosis and Are Associated with Higher Cardiovascular Risk Scores in Patients with NAFLD or Viral Chronic Liver Disease. Diagnostics.

[22] Abe M., Miyake T., Kuno A., Imai Y., Sawai Y., Hino K., Hara Y., Hige S., Sakamoto M., Yamada G. (2015). Association between Wisteria floribunda agglutinin-positive Mac-2 binding protein and the fibrosis stage of non-alcoholic fatty liver disease. J. Gastroenterol..

[23] Ogawa Y., Honda Y., Kessoku T., Tomeno W., Imajo K., Yoneda M., Kawanaka M., Kirikoshi H., Ono M., Taguri M. (2018). Wisteria floribunda agglutinin-positive Mac-2-binding protein and type 4 collagen 7S: Useful markers for the diagnosis of significant fibrosis in patients with non-alcoholic fatty liver disease. J. Gastroenterol. Hepatol..

[24] Yoneda M., Mawatari H., Fujita K., Yonemitsu K., Kato S., Takahashi H., Kirikoshi H., Inamori M., Nozaki Y., Abe Y. (2007). Type IV collagen 7s domain is an independent clinical marker of the severity of fibrosis in patients with nonalcoholic steatohepatitis before the cirrhotic stage. J. Gastroenterol..

[25] Ampuero J., Pais R., Aller R., Gallego-Durán R., Crespo J., García-Monzón C., Boursier J., Vilar E., Petta S., Zheng M.H. (2020). Development and Validation of Hepamet Fibrosis Scoring System–A Simple, Noninvasive Test to Identify Patients With Nonalcoholic Fatty Liver Disease With Advanced Fibrosis. Clin. Gastroenterol. Hepatol..

[26] Ballestri S., Nascimbeni F., Baldelli E., Marrazzo A., Romagnoli D., Targher G., Lonardo A. (2017). Ultrasonographic fatty liver indicator detects mild steatosis and correlates with metabolic/histological parameters in various liver diseases. Metabolism.

[27] Xavier S.A., Monteiro S.O., Arieira C.M., Castro F.D., Magalhães J.T., Leite S.M., Marinho C.M., Cotter J.B. (2021). US-FLI score—Is it possible to predict the steatosis grade with an ultrasonographic score?. Mol. Genet. Metab..

[28] (2018). The National Health and Nutrition Survey in Japan. https://www.mhlw.go.jp/content/000681200.pdf.

[29] Tanaka J., Koyama T., Mizui M., Uchida S., Katayama K., Matsuo J., Akita T., Nakashima A., Miyakawa Y., Yoshizawa H. (2011). Total numbers of undiagnosed carriers of hepatitis C and B viruses in Japan estimated by age- and area-specific prevalence on the national scale. Intervirology.

[30] Colli A., Pozzoni P., Berzuini A., Gerosa A., Canovi C., Molteni E.E., Barbarini M., Bonino F., Prati D. (2010). Decompensated chronic heart failure: Increased liver stiffness measured by means of transient elastography. Radiology.

